# Alterations of structural connectivity and structural co-variance network in focal cortical dysplasia

**DOI:** 10.1186/s12883-021-02358-7

**Published:** 2021-08-27

**Authors:** Dong Ah Lee, Ho-Joon Lee, Hyung Chan Kim, Kang Min Park

**Affiliations:** 1 Neurology Department, Haeundae Paik Hospital, Inje University College of Medicine, Haeundae-ro 875, Haeundae-gu, 48108 Busan, Korea; 2 Radiology Department, Haeundae Paik Hospital, Inje University College of Medicine, Busan, Korea

**Keywords:** Focal cortical dysplasia, Magnetic resonance imaging, Epilepsy

## Abstract

**Background:**

The aim of this study was to investigate alterations in structural connectivity and structural co-variance network in patients with focal cortical dysplasia (FCD).

**Methods:**

We enrolled 37 patients with FCD and 35 healthy controls. All subjects underwent brain MRI with the same scanner and with the same protocol, which included diffusion tensor imaging (DTI) and T1-weighted imaging. We analyzed the structural connectivity based on DTI, and structural co-variance network based on the structural volume with T1-weighted imaging. We created a connectivity matrix and obtained network measures from the matrix using the graph theory. We tested the difference in network measure between patients with FCD and healthy controls.

**Results:**

In the structural connectivity analysis, we found that the local efficiency in patients with FCD was significantly lower than in healthy controls (2.390 vs. 2.578, *p* = 0.031). Structural co-variance network analysis revealed that the mean clustering coefficient, global efficiency, local efficiency, and transitivity were significantly decreased in patients with FCD compared to those in healthy controls (0.527 vs. 0.635, *p* = 0.036; 0.545 vs. 0.648, *p* = 0.026; 2.699 vs. 3.801, *p* = 0.019; 0.791 vs. 0.954, *p* = 0.026, respectively).

**Conclusions:**

We demonstrate that there are significant alterations in structural connectivity, based on DTI, and structural co-variance network, based on the structural volume, in patients with FCD compared to healthy controls. These findings suggest that focal lesions with FCD could affect the whole-brain network and that FCD is a network disease.

## Background

Focal cortical dysplasia (FCD) belongs to the spectrum of malformations of cortical development, characterized by deranged neurons in the white matter, dyslamination, and abnormal balloon cells, first described by Taylor et al. in 1971 [1, 2]. Although the pathomechanism underlying FCD has not been defined, environmental factors, such as perinatal injury or viral infections, and genetic factors are believed to disrupt formation of cortex in utero, which results in FCD [1]. Multiple studies support the hypothesis that post-migrational insult to the developing cerebral cortex results in FCD type I [1]. The histopathological features of FCD type II and its association with mutations in genes of the mammalian target of rapamycin (mTOR) pathway suggest that circumscribed, early abnormality in cell proliferation is the underlying pathomechansim [1, 3].

FCD is one of the most common major congenital malformations in patients with epilepsy, with a prevalence of 48 % [4]. In addition, it is one of the most common causes of drug-resistant focal epilepsy [5]. Although 25–33 %% of patients with FCD respond well to anti-seizure medications (ASMs), treatment is predominantly surgical and depends on the identification of structural and functional lesions [6]. The complicated interaction between neurons, and imbalance in inhibitory and excitatory neurotransmitters are known mechanisms of epileptogenesis in FCD; with overexpression of AMPA and NMDA receptor subunits and loss of GABAergic neurons [7, 8].

Many studies have recently suggested that focal epilepsy is not a limited to a specific lesion, but results in alterations of the whole-brain network, due to the characteristic feature of the brain as a complex and interconnected system [9, 10]. FCD has been shown to exhibit a decrease in the volume and fractional anisotropy in certain white matter tracts [11]. However, little is known about the structural connectivity or network in patients with FCD. Graph theory is a branch of mathematics that represents a network as elements and their pairwise interconnections, also called nodes and edges [12]. Graph theory can summarize a complex network in the simplest form, as a connection matrix. This can offer important new insights into the structure of the networked brain systems [12]. Structural connectivity can be constructed by using diffusion tensor imaging (DTI), in which the fiber count obtained by accessing fibers connecting each possible pair of region of interests (ROIs) can be presented as a weighted matrix, the structural connectome [12, 13]. Structural connectivity is highly predictive and places constraints on the functional interactions across the brain network [14]. The study of structural connectivity is complemented by assessment of structural co-variance network, based on the regional structural volume or thickness. Structural co-variance network analysis can detect manifestation of persistent functional-trophic crosstalk, maturational changes, and common developmental and pathological influences [10]. Based on the previous researches regarding FCD, [15–19] we can predict that the global structural connectivity or co-variance network in patients with FCD have decreased integration and segregation in brain network topology.

In this study, we investigated the alterations in structural connectivity based on DTI and structural co-variance network based on the structural volume, using graph theory, in patients with FCD, compared to healthy controls. We hypothesized that focal lesions with FCD could affect and cause many changes in the whole-brain network. In addition, we analyzed the differences in structural connectivity and structural co-variance network between FCD patients with frontal lobe epilepsy and those with temporal lobe epilepsy.

## Methods

### Participants

**Fig. 1:**
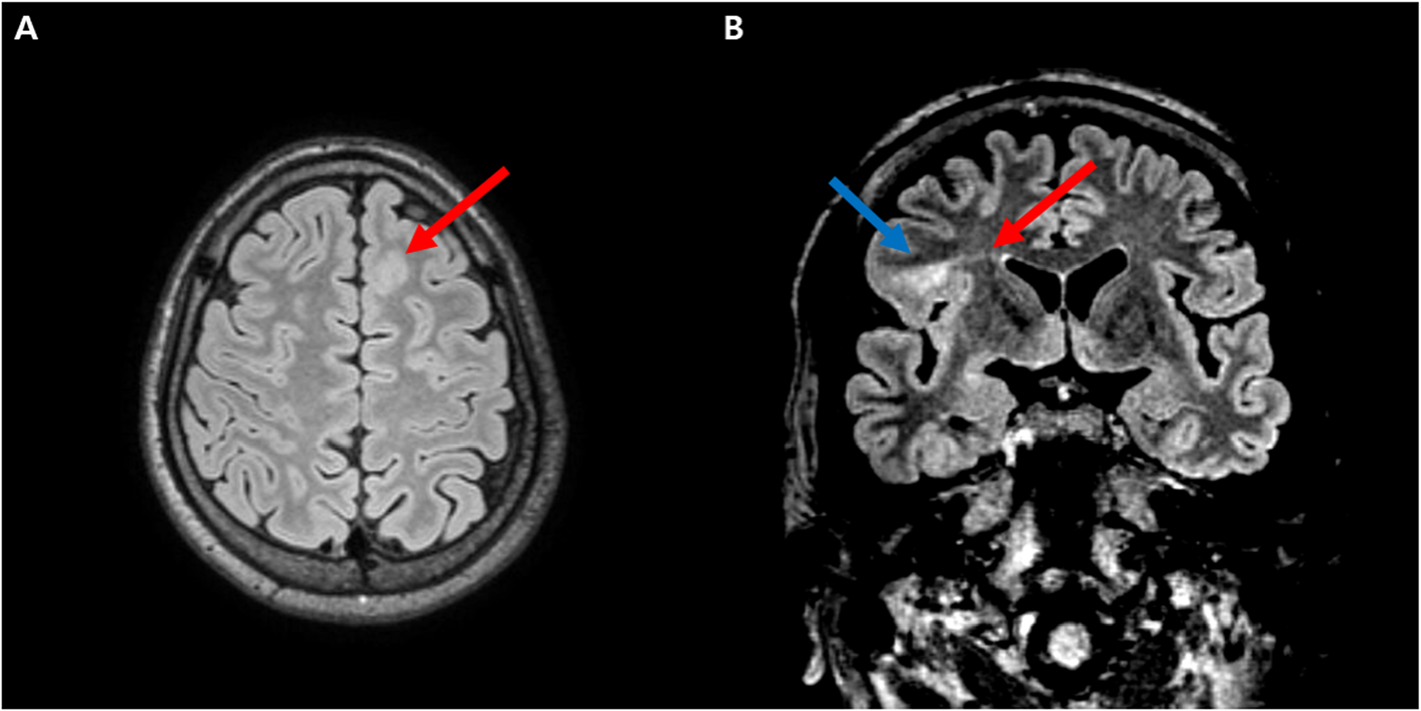
An example of typical brain MRI findings compatible with a focal cortical dysplasia. Fluid-attenuated inversion recovery (FLAIR) MRI of a patient showing focal cortical thickening with blurring of gray-white matter differentiation in the left frontal lobe (red arrow) (**A**). FLAIR MRI of another patient reveals focal increased signal intensity (blue arrow) with transmantle sign (red arrow) in the right frontal lobe (**B**)

This study was approved by the Institutional Review Board of our center (Number: 2020-08-009-003). Thirty-seven patients with FCD were retrospectively recruited at an epilepsy center in a tertiary hospital between March 2010 and December 2020. All patients presented with seizures and were diagnosed with epilepsy. The clinical diagnosis of FCD was based on the concordance between seizure semiology, electroencephalography (EEG), brain magnetic resonance imaging (MRI) findings, recurrent stereotyped seizures, and focal interictal or ictal EEG epileptiform discharges that coincided with an FCD-concordant lesion on MRI. The relevant brain MRI features included abnormal gyral pattern, increased cortical thickness, transmantle sign, and blurring of the gray matter-white matter interface (Fig. 1) [5, 20, 21]. None of the patients had any other structural lesions on brain MRI, except FCD. We investigated the clinical characteristics of patients with FCD at the time of MRI, such as age, sex, age at seizure onset, duration of epilepsy, and ASM load (calculated by the defined daily dose of the World Health Organization (WHO) [22].

In addition, we enrolled 35 age- and sex-matched healthy controls, who had no history of medical, neurological, or psychiatric problems. The subjects in the control group showed normal brain MRI findings.

### MRI scans

All subjects, patients with FCD as well as healthy controls, underwent brain MRI with the same scanner and protocols. MRI scans were performed using a 3.0T MRI scanner (AchievaTx, Phillips Healthcare, Best, The Netherlands) equipped with a 32-channel head coil. All subjects underwent brain MRI, as follows: 3-dimensional (3D) fluid-attenuated inversion recovery (FLAIR), coronal T2-weighted imaging, 3D T1-weighted imaging, and DTI. 3D T1-weighted images were obtained using a turbo-field echo sequence (TI = 1300 ms, repetition time/echo time [TR/TE] = 8.6/3.96 ms, flip angle = 8°, and 1 mm^3^ isotropic voxel size). DTI was performed using spin-echo single-shot echo-planar pulse sequences in 32 different diffusion directions (TR/TE = 8620/85 ms, flip angle = 90°, slice thickness = 2.25 mm, acquisition matrix = 120 × 120, field of view = 240 × 240 mm^2^, and *b*-value = 1,000 s/mm^2^).

### Structural connectivity and structural co-variance network analysis using graph theory

**Fig. 2:**
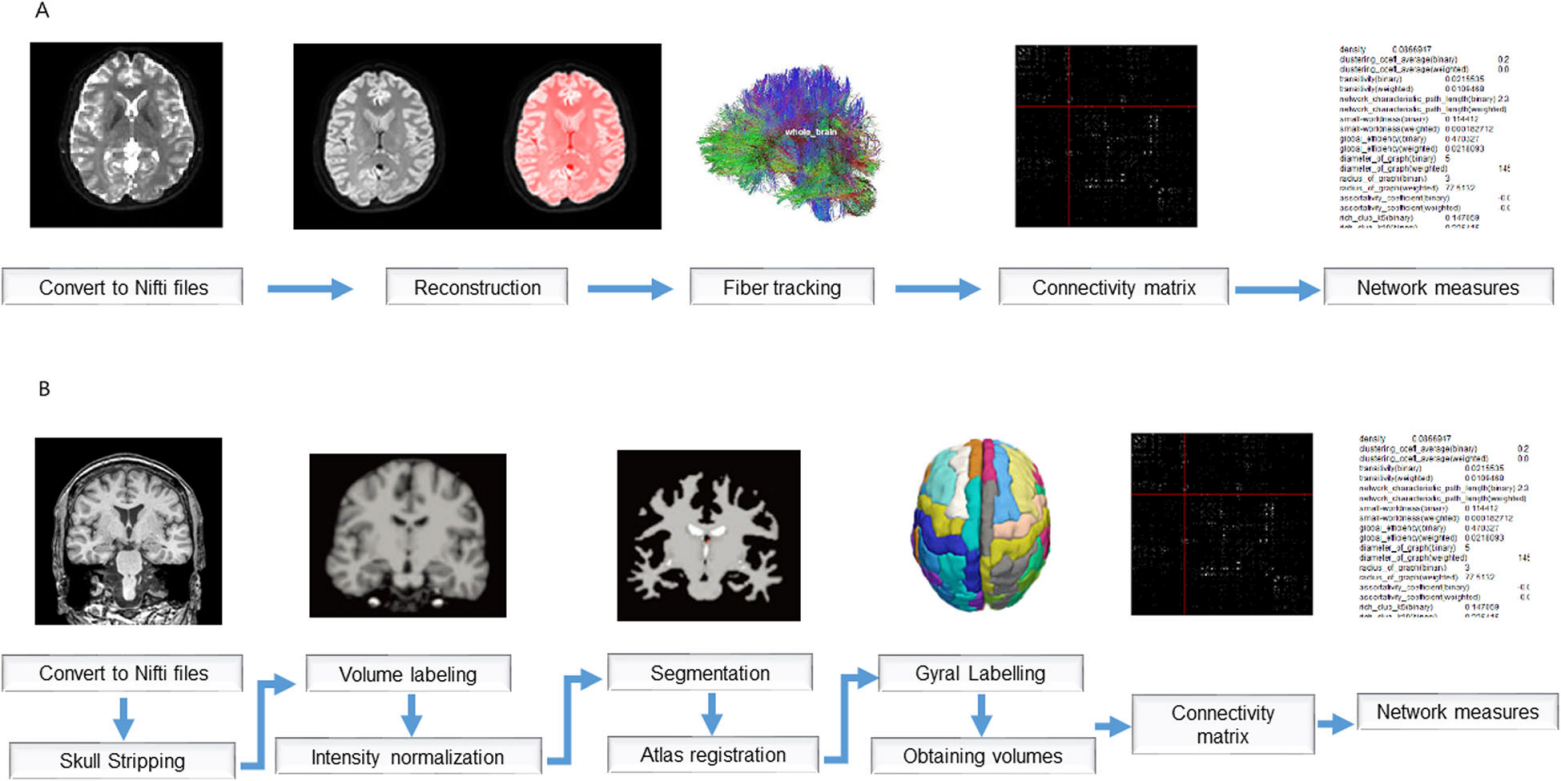
The process of graph theoretical analysis of the structural connectivity based on diffusion tensor imaging (**A**) and the structural co-variance network based on the structural volume with T1-weighted imaging (**B**)

We analyzed the structural connectivity with DTI in patients with FCD and healthy controls using a DSI studio program (Fig. 2 A). Initially, we read DTI raw images as a DICOM format. Then, we checked and corrected the eddy current distortion and motion artifact of images. We setup a mask, to filter out the background region, increase the reconstruction efficacy, and facilitate further visualization, with a process of thresholding, smoothing, and defragment. We conducted the data reconstruction using DTI method. We subsequently tracked the fibers with a deterministic fiber tracking algorithm. A seeding region was placed at whole brain and a total of 50,000 seeds were placed. The tracks with length shorter than 20 or longer than 300 mm were discarded. HCP842 tractography was used as the brain parcellation, and the connectivity matrix was calculated by using count of the connecting tracks. Finally, we extracted the network measures from the matrix, such as the mean clustering coefficient, characteristic path length, global efficiency, local efficiency, small-worldness index, transitivity, radius of graph, diameter of graph, and assortative coefficient, using graph theory [12, 23].

We calculated the structural co-variance network based on structural volume with 3D T1-weighted imaging using FreeSurfer and BRAPH programs (Fig. 2B) [23]. The analysis method has been described in detail in our earlier studies [24, 25]. Briefly, we obtained the structural volume of 81 ROIs using a FreeSurfer cortical reconstruction process. We used the order of “recon-all”. We then created an undirectional weighted connectivity matrix, in which the node was defined as the volume of the ROIs, and the edge was defined as the partial correlation among the volume of ROIs corrected for age and sex. Finally, we extracted the network measures from the matrix using graph theory. When we made a connectivity matrix in the comparison of structural co-variance network between FCD patients with frontal lobe epilepsy and those with temporal lobe epilepsy, we used covariates including age, sex, duration of epilepsy, and ASM load.

### Statistical analysis

We tested the statistical significance of the differences in structural connectivity between patients with FCD and healthy controls using independent Student’s t-test and between FCD patients with frontal lobe epilepsy and those with temporal lobe epilepsy using analysis of covariance with covariates including duration of epilepsy and ASM load (with a MedCalc program, version 19.6.4). Furthermore, we tested the statistical significance of the differences in structural co-variance network using a nonparametric permutation test (with a BRAPH program) between the two groups [26] because we could obtain the network measures at the group level. Statistical significance was set at *p* < 0.05. We applied multiple corrections using a false discovery rate method in connectivity analysis, and we represented corrected *p*-value.

Categorical variables are presented as number and percentile, and continuous variables as mean value and standard deviation (with normal distribution) or median value with interquartile range (without normal distribution).

## Results

### Clinical characteristics

The mean age of 37 patients with FCD was 28 years, and approximately half of them were male (54 %). The most common FCD location was the frontal lobe. Of the 37 patients, only six patients were pathologically confirmed FCD. Table 1 shows the clinical characteristics of the patients with FCD and healthy controls. Age and sex were not significantly different between patients with FCD and healthy controls.

**Table 1:** Clinical characteristics of patients with focal cortical dysplasia and healthy controls

	Patients with focal cortical dysplasia (*N* = 37)	Healthy controls (*N* = 35)	*p*-value
Age, years (± SD)	28.0 (± 9.8)	25.5 (± 2.6)	0.137
Male, n (%)	20 (54.0)	21 (60.0)	0.613
Age of seizure onset, years (interquartile range)	14 (11–23)		
Duration of epilepsy, months (interquartile range)	78 (12–180)		
EEG epileptiform discharges, n (%)	37 (100.0)		
ASM load (interquartile range)	1.4 (0.6–2.5)		
FCD side			
Right side, n (%)	16 (43.2)		
Left side, n (%)	20 (54.1)		
Both sides, n (%)	1 (2.7)		
FCD Location			
Frontal lobe, n (%)	16 (43.2)		
Temporal lobe, n (%)	15 (40.5)		
Parietal lobe, n (%)	3 (8.1)		
Occipital lobe, n (%)	3 (8.1)		
Transmantle sign, n (%)	10 (27.0)		

*SD* standard deviation; *EEG* electroencephalography; *ASM* anti-seizure medication; *FCD* focal cortical dysplasia

### Structural connectivity

Table 2 shows the differences in the structural connectivity between patients with FCD and healthy controls. There was a significant difference in structural connectivity between them. The local efficiency in patients with FCD was significantly lower than the healthy controls (2.390 vs. 2.578, *p* = 0.031). However, other network measures of structural connectivity, such as mean clustering coefficient, characteristic path length, global efficiency, small-worldness index, transitivity, radius of graph, diameter of graph, and assortative coefficient, were not different between patients with FCD and healthy controls.

**Table 2:** Differences in the structural connectivity using diffusion tensor imaging between patients with focal cortical dysplasia and healthy controls

Network measures	Patients with focal cortical dysplasia (*N* = 37)		Healthy controls (*N* = 35)			
	**Mean**	**SD**	**Mean**	**SD**	**Difference**	***p*-value**
Mean clustering coefficient	0.218	0.077	0.221	0.098	0.003	0.872
Characteristic path length	4.419	0.485	4.380	0.416	-0.039	0.719
Global efficiency	1.524	0.178	1.573	0.154	0.049	0.221
Local efficiency	2.390	0.348	2.578	0.376	0.187	0.031
Small-worldness index	0.237	0.098	0.248	0.119	0.010	0.685
Transitivity	0.259	0.093	0.251	0.109	-0.008	0.742
Radius of graph	1.712	0.318	1.656	0.236	-0.056	0.399
Diameter of graph	3.227	0.605	3.097	0.407	-0.130	0.293
Assortative coefficient	0.167	0.100	0.139	0.122	-0.027	0.299
**Network measures**	**Patients with frontal lobe epilepsy (*N* = 16)**		**Patients with temporal lobe epilepsy (*N* = 15)**			
	**Mean**	**SD**	**Mean**	**SD**	**Difference**	***p*-value**
Mean clustering coefficient	0.226	0.080	0.205	0.080	-0.021	0.459
Characteristic path length	4.453	0.454	4.224	0.366	-0.228	0.135
Global efficiency	1.504	0.186	1.572	0.182	0.067	0.316
Local efficiency	2.320	0.378	2.475	0.329	0.154	0.234
Small-worldness index	0.246	0.102	0.229	0.105	-0.016	0.652
Transitivity	0.280	0.098	0.240	0.095	-0.039	0.263
Radius of graph	1.763	0.406	1.663	0.243	-0.099	0.418
Diameter of graph	3.307	0.783	3.126	0.436	-0.180	0.437
Assortative coefficient	0.152	0.113	0.164	0.092	0.011	0.764

*SD* standard deviation

There were no differences in structural connectivity between FCD patients with frontal lobe epilepsy and those with temporal lobe epilepsy.

### Structural co-variance network

Table 3 presents the differences in the structural co-variance network based on the volume between patients with FCD and healthy controls. There were significant differences in the structural co-variance network between them. The mean clustering coefficient, global efficiency, local efficiency, and transitivity in patients with FCD were significantly lower than in the healthy controls (0.527 vs. 0.635, *p* = 0.036; 0.545 vs. 0.648, *p* = 0.026; 2.699 vs. 3.801, *p* = 0.019; 0.791 vs. 0.954, *p* = 0.026, respectively). However, the other network measures of structural co-variance network, such as characteristic path length, small-worldness index, radius of graph, diameter of graph, and assortative coefficient, did not differ between patients with FCD and healthy controls.

**Table 3:** Differences in the structural co-variance network based on the volume between patients with focal cortical dysplasia and healthy controls

Network measures	Patients with focal cortical dysplasia (*N* = 37)	Healthy controls (*N* = 35)				
	**Mean**	**Mean**	**Difference**	**CI lower**	**CI upper**	***p*-value**
Mean clustering coefficient	0.527	0.635	0.108	-0.105	0.100	0.036
Characteristic path length	1.968	1.651	-0.317	-0.323	0.286	0.051
Global efficiency	0.545	0.648	0.102	-0.083	0.092	0.026
Local efficiency	2.699	3.801	1.102	-0.893	0.894	0.019
Small-worldness index	0.984	0.984	-0.001	-0.019	0.018	0.481
Transitivity	0.791	0.954	0.163	-0.144	0.135	0.026
Radius of graph	2.539	2.632	0.093	-1.034	1.117	0.479
Diameter of graph	4.059	4.386	0.327	-1.779	1.804	0.367
Assortative coefficient	-0.013	-0.014	-0.001	-0.012	0.010	0.424
**Network measures**	**Patients with frontal lobe epilepsy (*N* = 16)**	**Patients with temporal lobe epilepsy (*N* = 15)**				
	**Mean**	**Mean**	**Difference**	**CI lower**	**CI upper**	***p*-value**
Mean clustering coefficient	0.627	0.470	-0.157	-0.173	0.171	0.081
Characteristic path length	1.656	2.175	0.518	-0.522	0.563	0.067
Global efficiency	0.646	0.520	-0.126	-0.136	0.141	0.094
Local efficiency	3.795	2.416	-1.379	-1.434	1.479	0.084
Small-worldness index	0.982	0.957	-0.025	-0.040	0.035	0.160
Transitivity	0.941	0.714	-0.227	-0.254	0.226	0.073
Radius of graph	2.343	3.128	0.784	-1.072	1.173	0.149
Diameter of graph	3.515	4.823	1.307	-2.028	2.150	0.192
Assortative coefficient	-0.015	-0.037	-0.022	-0.023	0.018	0.052

*CI* 95 % confidence interval of difference between the groups

There were no differences in structural co-variance network between FCD patients with frontal lobe epilepsy and those with temporal lobe epilepsy.

## Discussion

In this study, we found that there were significant alterations in structural connectivity and structural co-variance network in patients with FCD compared to healthy controls. These findings suggest that focal lesions in FCD can produce alterations in the whole-brain network. However, there were no differences in structural connectivity and structural co-variance network between FCD patients with frontal lobe epilepsy and those with temporal lobe epilepsy.

The present findings are in agreement with previous studies that have investigated the brain network in FCD using various MRI modalities. Liu et al. investigated the functional connectivity using resting state functional MRI and graph theory, and they found disrupted interactions and dysconnectivity in a large-scale neural network in patients with FCD, compared to healthy controls [15]. Another study analyzed the functional connectivity using magnetoencephalography recordings and graph theory, and successfully demonstrated that the brain had increased functional connectivity in the beta and gamma frequency bands in resting state in patients with FCD compared to healthy controls. [16]. In addition, a graph theoretical analysis of structural co-variance network using the cortical thickness of ROIs has indicated a consistent rearrangement characterized by inefficient global and excessive local connectivity in patients with FCD [17]. Interestingly, another study using DTI showed that the structural connectivity in FCD patients with frontal lobe epilepsy was more severe than those with temporal lobe epilepsy, suggesting different brain network disruptions according to FCD location [18]. Our study is the first to investigate the alterations in structural connectivity based on DTI and structural co-variance network based on volume in patients with FCD and compare it to healthy controls, and successfully demonstrate significant changes in the whole-brain network.

In the structural connectivity analysis, we found that the local efficiency in patients with FCD was lower than in healthy controls. Structural co-variance network analysis revealed that the mean clustering coefficient, global efficiency, local efficiency, and transitivity were lower in patients with FCD than in healthy controls. The local efficiency was calculated as the inverse of the average shortest path connecting the given node with all other nodes, which provides a measure of the efficiency of a given node in communicating with the rest of the brain [27]. The mean clustering coefficient was calculated as the mean local clustering coefficient, averaged over all nodes in the network, which assesses the degree to which the regions cluster, providing a measure of local connectivity [27]. The global efficiency was calculated as the average of the inverse of the shortest path length in a network, which estimates the ability of the network for parallel information transfer [27]. Decreased local efficiency and mean clustering coefficient reflects a decrease of segregation in a network, and decreased global efficiency represents a decrease of integration in a network. Segregation is supported by densely connected network communities, whereas integration is promoted by network hubs that are rich in connections between the communities, referred to as the ‘rich club’, members of which have high graphical measures of node degree and betweenness [28]. Thus, decreased segregation and integration decreases small-worldness in a network, which plays a crucial role in complex dynamical processes such as information transmission, pattern recognition, or learning [29]. These findings suggest decreased connectivity of the whole-brain network in patients with FCD.

Both segregation and integration of a brain network are critical for cognitive function [28, 30]. Thus, their alteration can be related to cognitive dysfunction, behavioral issues, or developmental delay, which are the clinical presentations of patients with FCD [20, 31]. Furthermore, previous studies have indicated a high prevalence of autism spectrum disorders in patients with FCD [32]. In connectivity studies, it has been demonstrated that autism spectrum disorders are accompanied by abnormal functional and structural features in specific brain regions of the default mode network [33]. Thus, we can postulate that all these disorders lie in a continuum of network diseases.

Recently, there have been studies showing the usefulness of connectivity analysis in clinical practice. We previously demonstrated that the assortative coefficient, one of the network measures, differed according to the ASM response among patients with newly diagnosed focal epilepsy, which suggested that the changes in brain connectivity could be a potential biomarker for predicting the response to ASM [34]. Another study revealed the potential use of brain connectivity as a predictor for surgical outcome in epilepsy. Bi-hemispheric alterations of thalamotemporal structural networks represented a poor surgical outcome in temporal lobe epilepsy [35]. From these previous researches, we could assume that investigating the associations between medical/surgical outcomes and brain connectivity in patients with FCD can be an interesting topic in future studies.

Although we successfully demonstrate alterations in structural connectivity and structural co-variance network in patients with FCD, there were several limitations to this study. First, we included patients with FCD with a clinical presentation of seizures. Thus, we cannot exclude the possibility that alterations in structural connectivity and structural co-variance network in patients with FCD might have been caused by symptoms of seizures or ASMs. A previous study demonstrated the significant effects of ASMs on the brain network [36]. However, it was difficult to enroll patients with FCD who had no neurological or psychiatric problems. Second, we only looked at the structural connectivity and structural co-variance network at the whole-brain network level, because the specific location of FCD was variable. Thus, we could not investigate whether decreased connectivity was related to the location of FCD lesion. In addition, heterogeneity of FCD lesion, including varying size and location hampered the assessments in network analysis and produced confounding effects on the results. Lastly, the diagnosis of most of the patients was not pathologically confirmed, but were diagnosed with FCD based on clinical and MRI findings. However, we exclusively enrolled patients with typical MRI findings, compatible with FCD. Further studies with pathologically confirmed patients in a large sample size may be needed to confirm our findings.

## Conclusions

We demonstrate that there are significant alterations in structural connectivity based on DTI and structural co-variance network based on volume in patients with FCD compared to healthy controls. These findings suggest that focal lesions with FCD could affect the whole-brain network, and additionally, that FCD is a network disease.

## Data Availability

The datasets used and/or analyzed during the current study are available from the corresponding author on reasonable request.
